# Editorial: World health day 2022: Impact of COVID-19 on health and socioeconomic inequities

**DOI:** 10.3389/fpubh.2023.1180628

**Published:** 2023-03-28

**Authors:** Martin Amogre Ayanore, Amanda Rodrigues Amorim Adegboye, Hubert Amu, Chandan Kumar, Rasheda Khanam

**Affiliations:** ^1^Department of Health Policy Planning and Management, Fred N. Binka School of Public Health, University of Health and Allied Sciences, Ho, Ghana; ^2^Centre for Agroecology, Water and Resilience, Coventry University, Coventry, United Kingdom; ^3^Centre for Healthcare Research, Coventry University, Coventry, United Kingdom; ^4^Department of Population and Behavioral Sciences, Fred N. Binka School of Public Health, University of Health and Allied Sciences, Ho, Ghana; ^5^Department of Policy and Management Studies, TERI School of Advanced Studies (TERI SAS), New Delhi, India; ^6^School of Business, University of Southern Queensland, Toowoomba, QLD, Australia

**Keywords:** World health day 2022, COVID-19, socioeconomic inequalities and health, Sustainable Development Goals (SDGs), equity in health

Growing health and socioeconomic inequalities within and across countries are a defining challenge of our times and remain a huge obstacle to the realization of the United Nations Sustainable Development Goals (SDGs) (1). Overall, COVID-19 has revealed a pandemic of inequality, including access to vaccines in many settings in the world (2).

Building resilient health systems post-COVID-19 will require identifying cost-effective interventions, employing a health system learning approach that assists us build-back what was lost in the pandemic, and providing opportunities to address health inequalities. Overall, there is a need for policy-oriented research that reveal how health emergencies, such as the COVID-19 pandemic exacerbates and expose existing inequalities in society (3).

Given the impact of the COVID-19 pandemic on global affairs, we present and highlight the impact of the pandemic on health and socioeconomic inequities in this Research Topic. Since WHO's founding in 1948, it has led the way in advancing and promoting health across the world; from post-world world II recovery efforts to advance health to the Alma Atta declaration, to the enactment of the Millennium Development Goals (MDGs) in 2000 and now the SDGs from 2015-2030 (4).

In this collection, we accepted and published nine original research and one opinion piece. The ten publications are classified into four domains; quality of life pre/post the pandemic, COVID-19, and welfare loss (economic social, and human), COVID-19 and social trust for pandemic control, and clinical and technological innovations for enhancing health systems for current and future pandemics.

First, two studies in the Chinese population examined social and economic factors that influenced health related quality of life (HRQoL). These studies show that social disruptions brought about during pandemics shape QoL outcomes. Specifically, welfare forces such as job change, and family conflicts due to the pandemic decreased the quality of life (QoL). Other studies in other context have highlighted job and wage losses and changes in metal and psychosocial health as social disruptions due to COVID-19. Liu et al. established that the interaction effects of job changes and family conflict on QoL were significant among fathers and one-child families, highlighting the crucial role of social disruptions at the family structure level, during pandemics.

On the other hand, Wong et al. found that loneliness exacerbated by the pandemic influenced poor QoL outcomes, particularly among older adults.

During pandemics, social and economic disruptions impact on mental health and wellbeing (5, 6). In addition, the existence of a pandemic may cause low priority actions for local epidemics, as the case in Ghana (7) during the first wave of the COVID-19 pandemic when health systems were yet adjusting to deal with the pandemic. To address QoL inequalities during pandemics, social protection policies that identify and prioritize vulnerable groups and provide safety nets to meet social needs such as housing, loneliness, job, and welfare losses can assist address psychosocial health needs and bridge health inequalities in the population.

Second, COVID-19 manifested economic, social, and human losses. Alenzi et al. from their study found the economic burden of COVID-19 to have impacted negatively on households and the health system, particularly for the vulnerable. During health emergencies, increased demand and pressure on health systems raise both direct and indirect healthcare costs, with implications for poor and vulnerable groups' access to healthcare services. In instances where catastrophic payments are experienced, inequalities are further deepened in society.

Leung et al. also reported from their study that socioeconomic disparities of perceived benefits and harms existed in Saudi Arabia amid the pandemic. To address socioeconomic disparities and reduce economic burden during emergencies, policy actions that address financial risk protection and reduce disparities in access to social care services are vital for long-term progress.

During pandemics, data science can play a significantly role by providing context evidence for localized pandemic control actions, as exemplified by Manz et al. in Germany. For example, in deprived areas in Bavaria, Germany, the estimation of standardized incidence and mortality ratios allowed for deeper understanding of disease burden in deprived districts. This application for data science is vital for targeting during pandemics, particularly population groups that are worse off or disadvantaged in a pandemic. Aside human and social disruptions, the pandemic affected business and supply chain systems globally, with negative repercussions on world economies as reported by Zhao. Despite the negative effects of the pandemic on business enterprises, a small window of opportunity was also presented for innovation as highlighted by Li et al., however, this evidence remains scanty and not uniform. Future studies need to explore the wealth or opportunity that pandemics present for business innovations in many contexts.

Third, building strong social trust for pandemic control highlight the construct of citizen trust in governments and scientist as vital in the management of the COVID-19 pandemic as reported by Bajos et al.. Trust allows a person with low knowledge and power to make decisions that align with their wellbeing and is historically a very important component of healthcare (8). The study highlights the urgent need for pandemic response strategies to include depoliticized approaches that target disadvantaged groups while ensuring social inclusion in risk communication and vaccine programs.

Lastly, the pandemic witnessed a surge in digital innovations in the health industry. Specht et al. illustrated how digital innovations during the pandemic provided an opportunity to address homeless and vulnerable healthcare needs. During the early period of the first doses of COVID-19 vaccines, data show the Gini coefficients for COVID-19 vaccines were 0.91 and 0.88 on June 7 and December 7, 2021, respectively while between June 7 and December 7, 2021, the Gini coefficients were 0.57 and 0.61, respectively, indicating severe inequality thresholds in all cases (9).

Inequality in vaccine distribution was attributed to economic, financial support and human factors, infrastructure, and health system, legal and political, epidemiologic and demographic factors (10). The opinion expressed by Saleh shows the need for LMIC to invest in Research and Development (R&D) through the active establishment and promotion of clinical trials as an entry point to address future vaccine inequalities and address specific population groups' access to equitable healthcare. Such a bold and decisive step will ensure LMIC and HIC health systems adopt a health system learning approach to context health problems.

In conclusion, our Research Topic highlights pathways where inequalities were exacerbated during the pandemic. COVID-19 has highlighted the relevance of policy actions that address broadly social determinants of health to mitigate the impacts of future pandemics on health inequalities. The Research Topic also emphasizes the relevance of digital health and technology in addressing health equity goals, particularly the SDGs. Future studies on randomized control trials and those that enable causality relationships to be established are further encouraged.

## Author contributions

MA conceptualized and wrote the initial draft. HA reviewed first draft. AA, CK, and RK reviewed the revised draft and made inputs into the final draft. All authors read and approved the manuscript for submission.
